# New indicators for delay in initiation of antiretroviral treatment: estimates for Cameroon

**DOI:** 10.2471/BLT.14.147892

**Published:** 2015-06-01

**Authors:** Jacques DA Ndawinz, Xavier Anglaret, Eric Delaporte, Sinata Koulla-Shiro, Delphine Gabillard, Albert Minga, Dominique Costagliola, Virginie Supervie

**Affiliations:** a INSERM, Sorbonne Universités, UPMC Univ Paris 06, UMR_S 1136, Institut Pierre Louis d’Epidémiologie et de Santé Publique, F-75013, Paris, France.; b INSERM, Université Bordeaux, Bordeaux, France.; c IRD, Université de Montpellier 1, Montpellier, France.; d Faculty of Medicine and Biomedical Sciences, University of Yaoundé, Yaoundé, Cameroon.; e Programme PACCI, Abidjan, Côte d’Ivoire.

## Abstract

**Objective:**

To propose two new indicators for monitoring access to antiretroviral treatment (ART) for human immunodeficiency virus (HIV); (i) the time from HIV seroconversion to ART initiation, and (ii) the time from ART eligibility to initiation, referred to as delay in ART initiation. To estimate values of these indicators in Cameroon.

**Methods:**

We used linear regression to model the natural decline in CD4+ T-lymphocyte (CD4+ cell) numbers in HIV-infected individuals over time. The model was fitted using data from a cohort of 351 people in Côte d’Ivoire. We used the model to estimate the time from seroconversion to ART initiation and the delay in ART initiation in a representative sample of 4154 HIV-infected people who started ART in Cameroon between 2007 and 2010.

**Findings:**

In Cameroon, the median CD4+ cell counts at ART initiation increased from 140 cells/μl (interquartile range, IQR: 66 to 210) in 2007–2009 to 163 cells/μl (IQR: 73 to 260) in 2010. The estimated average time from seroconversion to ART initiation decreased from 10.4 years (95% confidence interval, CI: 10.3 to 10.5) to 9.8 years (95% CI: 9.6 to 10.0). Delay in ART initiation increased from 3.4 years (95% CI: 3.1 to 3.7) to 5.8 years (95% CI: 5.6 to 6.2).

**Conclusion:**

The estimated time to initiate ART and the delay in ART initiation indicate that progress in Cameroon is insufficient. These indicators should help monitor whether public health interventions to accelerate ART initiation are successful.

## Résumé

**Objectif:**

Proposer deux nouveaux indicateurs pour évaluer l'accès au traitement antirétroviral (TAR) contre le virus de l'immunodéficience humaine (VIH): (i) le délai entre la séroconversion au VIH et le début du TAR, et (ii) le délai entre l'éligibilité au TAR et le début du TAR, appelé «délai écoulé avant le début du TAR». Estimer l'utilité de ces indicateurs au Cameroun.

**Méthodes:**

Nous avons utilisé une régression linéaire pour modéliser le déclin naturel au fil du temps du nombre de lymphocytes T CD4+ (cellule CD4+) chez les individus infectés par le VIH. Le modèle a été conçu à l'aide de données provenant d'une cohorte de 351 individus de Côte d'Ivoire. Nous l'avons utilisé pour estimer le délai entre la séroconversion et le début du TAR ainsi que le retard dans l’accès au TAR sur un échantillon représentatif de 4154 individus infectés par le VIH ayant commencé un TAR au Cameroun entre 2007 et 2010.

**Résultats:**

Au Cameroun, le taux médian de CD4+ au début du TAR est passé de 140 cellules 140 cellules/μl (intervalle interquartile, IQR: de 66 à 210) en 2007–2009 à 163 cellules/μl (IQR: de 73 à 260) en 2010. Le délai moyen estimé entre la séroconversion et le début du TAR est passé de 10,4 années (intervalle de confiance, IC, à 95%: de 10,3 à 10,5) à 9,8 années (IC à 95%: de 9,6 à 10,0). Le retard dans l’ accès au TAR est passé de 3,4 années (IC à 95%: de 3,1 à 3,7) à 5,8 années (IC à 95%: de 5,6 à 6,2).

**Conclusion:**

Les estimations du délai entre la séroconversion et le début du TAR et du retard dans l’accès au TAR indiquent que les progrès accomplis au Cameroun sont insuffisants. Ces indicateurs devraient aider à évaluer l'efficacité des interventions de santé publique visant à accélérer l’accès au TAR .

## Resumen

**Objetivo:**

Proponer dos nuevos indicadores para monitorear el acceso al tratamiento antirretroviral (TAR) para el virus de la inmunodeficiencia humana (VIH); (i) el tiempo desde la seroconversión del VIH al inicio del TAR, y (ii) el tiempo desde la elegibilidad del TAR al inicio del mismo, referido como retraso en el inicio del TAR. Estimar los valores de estos indicadores en Camerún.

**Métodos:**

Se utilizó la regresión lineal para modelizar la disminución natural de la cantidad de linfocitos T CD4+ (célula CD4+) en los individuos infectados por el VIH con el tiempo. El modelo se adaptó utilizando datos de una cohorte de 351 individuos en Côte d’Ivoire. Se utilizó el modelo para estimar el tiempo entre la seroconversión y el inicio del TAR y el retraso en el inicio del TAR en una muestra representativa de 4.154 individuos infectados por el VIH que iniciaron el TAR en Camerún entre 2007 y 2010.

**Resultados:**

En Camerún, la media del recuento de células CD4+ en el inicio del TAR creció de 140 células/μl (rango intercuartílico, RIC: de 66 a 210) en 2007-2009 a 163 células/μl (RIC: de 73 a 260) en 2010. El tiempo medio estimado desde la seroconversión hasta el inicio del TAR descendió de 10,4 años (intervalo de confianza, IC, del 95%: de 10,3 a 10,5) a 9,8 años (IC del 95%: de 9,6 a 10,0). El retraso en el inicio del TAR aumentó de 3,4 años (IC del 95%: de 3,1 a 3,7) a 5,8 años (IC del 95%: de 5,6 a 6,2).

**Conclusión:**

El tiempo estimado para iniciar el TAR y el retraso en el inicio del TAR indican que el progreso en Camerún es insuficiente. Estos indicadores deberían ayudar a monitorizar si las intervenciones de salud pública para acelerar el inicio del TAR son eficaces.

## ملخص

**الغرض:**

اقتراح مؤشرات جديدة لمراقبة الحصول على علاج باستخدام مضادات الفيروسات القهقرية (ART) لمقاومة فيروس عوز المناعة البشري (HIV)؛ (1) الفترة الزمنية منذ انقلاب تفاعلية مصل HIV (ظهور أجسام مضادة له) حتى بدء ART، و(2) الفترة الزمنية منذ التأهل لـ ART حتى البدء فيه، والمشار إليه بالتأخر في بدءART . وتقييم القيم التي تم التوصل إليها بفضل هذه المؤشرات في الكاميرون.

**الطريقة:**

استخدمنا أسلوب التحوف الخطي للوصول إلى نموذج للانخفاض الطبيعي في أعداد الخلايا اللمفية التائية من فئة CD4+ (خلايا فئة CD4+ ) في الأشخاص المصابين بمرض عوز المناعة البشري مع مرور الوقت. وكان النموذج مناسبًا للغرض بفضل استخدام بيانات تم الحصول عليها من مجموعة من الأشخاص يبلغ عددهم 351 شخصًا في كوت ديفوار. وقد استخدمنا هذا النموذج لتقدير الفترة بين انقلاب تفاعلية المصل وبدءART من ناحية وتقدير فترة التأخر في بدء ART من ناحية أخرى، وذلك في عينة تمثل المرضى تشمل 4154 مصابًا ممن بدأواART  في الكاميرون في الفترة بين 2007 و2010.

**النتائج:**

زاد في الكاميرون وسيط القيمة لتعداد الخلايا فئة CD4+ عند بدء ART من 140 خلية/ميكرو لتر (المدى الربيعي: 66 إلى 210) في الفترة من 2007 – 2009 إلى 163 خلية/ميكرو لتر (المدى الربيعي: 73 إلى 260) في عام 2010. وانخفض متوسط الفترة الزمنية منذ انقلاب تفاعلية المصل حتى بدءART من 10.4 عام (بنسبة أرجحية مقدارها 95%: 10.3 إلى 10.5) إلى 9.8 عام (بنسبة أرجحية مقدارها 95%:  9.6 إلى 10.0). وزاد التأخر في بدء ART من 3.4 عام (بنسبة أرجحية مقدارها 95%: 3.1 إلى 3.7) إلى 5.8 عام (بنسبة أرجحية مقدارها 95%: 5.6 إلى 6.2).

**الاستنتاج:**

تشير الفترة الزمنية المقدرة لبدء ART وللتأخر في بدء ART إلى أن التقدم في العلاج في الكاميرون لا يفي بالاحتياجات. ومن المفترض أن تساعد هذه المؤشرات على مراقبة إذا ما كانت التدخلات في مجال الصحة العامة للإسراع ببدء ART ناجحة بالفعل.

## 摘要

**目的:**

为监控人类免疫缺陷病毒 (HIV) 的抗逆转录病毒治疗 (ART) 情况而提出两个新指标；(i) 从 HIV 血清转化到 ART 开始的时间，和 (ii) 从 ART 合格到开始的时间，也称为 ART 开始时间延迟。 旨在评价喀麦隆境内这些指标值。

**方法:**

我们用线性回归法建立了 HIV 感染者体内的 CD4+ T 淋巴细胞（CD4+ 细胞）数在一段时间内呈自然递减的模型。 该模型拟合从科特迪瓦境内 351 名人员中采集的数据。 我们以在喀麦隆境内于 2007 年至 2010 年期间接受 ART 的 4154 名 HIV 患者为代表性样本，采用该模型来估计血清转化到 ART 开始的时间以及 ART 开始时间的延迟。

**结果:**

在喀麦隆境内，ART 开始时的 CD4+ 细胞计数中值从 2007 年至 2009 年的 cells/μl（四分位差：IQR： 66 至 210）增加至 2010 年的 163 cells/μl（IQR： 73 至 260）。从血清转化到 ART 开始的估计平均时间从 10.4 年（95% 置信区间，CI： 10.3 至 10.5）下降至 9.8 年（95% CI： 9.6 至 10.0）。 ART 的开始时间延迟从 3.4 年（95% CI： 3.1 至 3.7）增加至 5.8 年（95% CI： 5.6 至 6.2）。

**结论:**

开始 ART 的预计时间和 ART 开始时间的延迟表明喀麦隆境内并未取得足够的进步。 这些指标应有助于监控公共卫生干预措施是否能够成功加快 ART 的开始时间。

## Резюме

**Цель:**

Предложить два новых показателя для контроля доступности антиретровирусной терапии (АРВТ), предназначенной для лечения вируса иммунодефицита человека (ВИЧ): (i) время от сероконверсии ВИЧ до начала АРВТ и (ii) время с момента определения соответствия пациента критериям назначения АРВТ до начала терапии, называемое задержкой начала АРВТ. Оценить значения данных показателей в Камеруне.

**Методы:**

Было смоделировано естественное снижение с течением времени числа T-лимфоцитов CD4+ (клеток CD4+) у инфицированных ВИЧ при помощи линейной регрессионной модели. Модель корректировалась с использованием данных когорты из 351 человека в Кот-д’Ивуаре. Модель применялась для оценки временного отрезка между сероконверсией и началом АРВТ, а также задержки начала АРВТ в репрезентативной выборке из 4154 ВИЧ-инфицированных, начавших АРВТ в Камеруне в период между 2007 и 2010 гг.

**Результаты:**

В Камеруне медианное число клеток CD4+ в начале АРВТ увеличилось с 140 клеток/μл (межквартильный размах, МКР: 66–210) в 2007–2009 гг. до 163 клеток/μл (МКР: 73–260) в 2010 г. Выявленное среднее время от сероконверсии до начала АРВТ сократилось с 10,4 года (95% доверительный интервал, ДИ: 10,3–10,5) до 9,8 года (95% ДИ: 9,6–10,0). Задержка начала АРВТ увеличилась с 3,4 года (95% ДИ: 3,1–3,7) до 5,8 года (95% ДИ: 5,6–6,2).

**Вывод:**

Полученные значения времени начала АРВТ и задержки начала АРВТ указывают на недостаточный прогресс в Камеруне. Эти показатели должны помочь в оценке успешности мероприятий в области здравоохранения, направленных на ускорение начала АРВТ.

## Introduction

Early initiation of antiretroviral treatment (ART) is needed to reduce morbidity and mortality from human immunodeficiency virus (HIV) infection and to reduce HIV transmission.^1^^,^^2^ The CD4+ T-lymphocyte (CD4+ cell) threshold for ART eligibility in World Health Organization (WHO) guidelines have changed several times. In 2006, HIV-infected individuals with 200 CD4+ cells/μL or less were eligible for ART. In 2010, 350 cells/μL was the threshold. In 2013, the threshold was raised to 500 cells/μL, but the guidance was to prioritize individuals with advanced HIV disease or less than 350 cells/μL.^3^^–^^5^

These changes in ART eligibility coincided with rapid scaling-up of ART in low- and middle-income countries. In sub-Saharan Africa, the number of people receiving ART increased from 100 000 in 2003 to 9.0 million in 2013.^6^ Between 2006 and 2011, the median CD4+ cell count increased from 238 to 286 cells/μL at enrolment into care and from 125 to 185 cells/μL at ART initiation.^7^^,^^8^ Thus, many people living with HIV started ART below the WHO-recommended CD4+ cell count threshold, which indicates a gap between ART guidelines and reality.

Measuring this gap at the population level and its changes over time is important to effectively monitor and evaluate programmes aimed at reducing late ART initiation. Generally, the timeliness of ART initiation is assessed by analysing the distribution of CD4+ cell counts at ART initiation and access to ART is measured by ART coverage, defined as the number of individuals receiving ART among those eligible.^9^ However, these indicators do not quantify the time between becoming HIV-infected and the actual ART initiation nor do they measure how much time is lost between the moment – usually unobserved – when HIV-infected people reach the ART eligibility threshold and when they actually start receiving ART. Here, we propose two new population-level indicators to measure these times among HIV-infected people initiating ART: (i) the time from HIV seroconversion – i.e. the time at which the body produces detectable antibodies to HIV – to ART initiation; and (ii) the delay in ART initiation, i.e. the time between eligibility for ART and initiation of ART. To give an example, we estimated these indicators for Cameroon, where ART became freely available in May 2007 and the number of people living with HIV and receiving ART increased from 17 000 in 2005 to 132 000 by the end of 2013.^6^

## Methods

### Data sources

Our approach requires two data sets. First, data on CD4+ cell counts at ART initiation among individuals who are representative of the population of interest. Second, data on the natural history of the CD4+ cell count decline during HIV infection.

The first data set was obtained from a survey conducted in a representative sample of 55 HIV-care facilities in Cameroon. The design of this survey has been described elsewhere.^10^ Briefly, the medical records of 4154 people older than 15 years, living with HIV and who started ART in the month of October in the years 2007–2010 were reviewed to obtain sociodemographic and CD4+ cell count data, including gender, age at ART initiation, date of ART initiation and CD4+ cell count before ART initiation. Most participants were women (2829, 68.1%), and median age at ART initiation was 35 years (Table 1).

**Table 1:** Characteristics of people living with HIV who started antiretroviral treatment, and had a measure of CD4+ cell count at treatment initiation, Cameroon 2007–2010

Characteristic	People who started ART *n* = 4154
**Number of women (%)**	2829 (68.1)
**Median age at ART initiation, years (IQR)**	35.0 (29.0 to 43.0)
**Median CD4+ cell count at ART initiation, cells/μL (IQR)**	
2007–2009	
All	140 (66 to 210)
Women	147 (73 to 215)
Men	123 (53 to 198)
2010	
All	163 (73 to 260)
Women	170 (78 to 263)
Men	147 (69 to 239)

ART: antiretroviral therapy; HIV: human immunodeficiency virus; IQR: interquartile range.

Notes: People included in the study were older than 15 years and started ART in the month of October each year. The ART eligibility threshold was 200 cells/μL in 2007–2009 and 350 cells/μL in 2010.

The second data set can be obtained from an observational cohort including individuals with a known or reliably estimated date of HIV seroconversion and repeated measurements of the CD4 cell count over time. As there was no such cohort study in Cameroon, we used data from a cohort of blood donors who had documented seroconversion between donations in Côte d’Ivoire (ANRS 1220 Primo-CI study).^11^^,^^12^ Blood donors had been invited to participate if they met the following criteria: diagnosed with HIV during a blood donation; HIV-seronegative at the preceding donation; less than 36 months had elapsed since seroconversion. The date of seroconversion was estimated as the midpoint between the last negative and the first positive HIV test. We used data on 351 cohort participants in our analyses, with 3037 CD4+ cell count measurements before ART initiation or death; CD4+ cell counts obtained more than 10 years after seroconversion were discarded to avoid a selection bias for slow progressors. Median age at seroconversion was 28.8 years and 60.7% (213) were men (Table 2). 

**Table 2:** Characteristics of ANRS 1220 Primo-CI cohort participants in Côte d’Ivoire

Characteristic	Cohort participants *n* = 351
Number of women (%)	138 (39.3)
Median age at seroconversion, years (IQR)	28.8 (24.5 to 34.4)
Median time between last negative and first positive HIV test, months (IQR)	8.5 (3.4 to 20.3)
Median time between estimated date of seroconversion and cohort inclusion, months (IQR)	9.0 (4.9 to 18.9)
Median CD4+ cell count at cohort inclusion, cell/μL (IQR)	450 (313 to 612)
Median number of CD4+ cell count measurements (IQR)	8.0 (3.0 to 13.0)
Median time between two consecutive CD4+ cell count measurements, months (IQR)	6.0 (5.9 to 6.1)
Median cohort follow-up, years (IQR)	5.6 (3.6 to 8.2)

HIV: human immunodeficiency virus; IQR: interquartile range.

Data sources: ANRS 1220 Primo-CI.^11^^,^^12^

The median time between the last negative and first positive HIV test was 8.5 months and the median time from seroconversion to inclusion into the cohort was 9.0 months. The median CD4+ cell count at cohort inclusion was 450 cells/μL and the median number of CD4+ cell measurements per individual was eight.

Blood samples from the participants were collected at inclusion into the cohort and every six months thereafter to measure the CD4+ cell count.

### Analyses

The analysis was completed in three steps. First, we modelled the natural decline in CD4+ cell counts in HIV-infected individuals who had not yet received ART, using data from the seroconversion cohort. Second, we used the model to estimate the average time from seroconversion to eligibility for ART in this cohort. Third, we used the model and data on CD4+ cell counts at ART initiation in Cameroon to estimate the average time between seroconversion and ART initiation in Cameroon. Then, we calculated the delay in ART initiation in Cameroon by subtracting the average time between HIV seroconversion and ART eligibility from the average time between HIV seroconversion and ART initiation.

We first used a linear mixed model^13^ to describe the natural decline in CD4+ cell numbers in ART-naïve HIV-infected individuals with estimated dates of HIV seroconversion and repeated measurements of the CD4+ cell count over time. To take into account the correlation of repeated measurements in a given individual, the parameters were allowed to vary from one individual to another through a random intercept and slope. CD4+ cell count data were square-root transformed to normalize their distribution.^14^ The model equations are as follows:
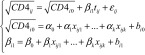
(1)where *CD*4*_ij_* is the CD4+ cell count measured at the *j*th visit of the *i*th individual, *CD*4*_i_*_0_ is the estimated CD4+ cell count at HIV seroconversion, defined as time 0 in the model; *β_i_*_1_ is the CD4+ cell count slope; *t_ij_* is the interval from the estimated date of seroconversion to the *j*th visit; and *ε_ij_* is the residual error.

Both the CD4+ cell count at seroconversion and the CD4+ slope may be influenced by covariates. *x_ijk_* represents the *k*th covariate; *α_k_* and *β_k_* are respectively the fixed-effect intercept and slope for the *k*th covariate; *α_0_* and *β_0_* are respectively the baseline intercept and slope; and *b_i0_* and *b_i_*_1_ are respectively the random intercept and slope for the *i*th individual.

*ε_ij_*, *b_i_*_0_ and *b_i_*_1_ were assumed to be normally distributed. The correlation between individual CD4+ cell counts at seroconversion and subsequent individual slopes was handled through an unstructured covariance matrix of random effects.

From Equation (1), we derived the time from seroconversion to any given CD4+ cell count:
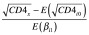
(2)where *CD*4*_x_* is a given CD4+ cell count, *E*(√CD4*_i_*_0_) and *E*(*β_i_*_1_) are expected values of √CD4*_i_*_0_ (the square root of the CD4+ cell counts at seroconversion) and *β_i_*_1_, (rate of CD4+ cell count decline) obtained by fitting Equation (1) to data from seroconverters.

Second, we used Equation (2), to calculate, for each seroconverter, the time from seroconversion to specified CD4+ count thresholds. From these individual times we calculated the average time from seroconversion to ART eligibility.

Third, we calculated the time from seroconversion to ART initiation, using Equation (2), we need to know the CD4+ cell count at ART initiation, the CD4+ cell count at HIV seroconversion and the average rate of CD4+ cell count decline. Many HIV-infected individuals starting ART have only one available CD4+ cell count before ART initiation, because they are already eligible for ART when HIV infection is diagnosed. Thus, the CD4+ cell count at seroconversion and the rate of CD4+ cell count decline are both unknown. We generated the values of these two parameters for each HIV-infected individual starting ART by using data from seroconverters.^11^^,^^12^ Specifically, for each individual included in the survey conducted in Cameroon, we simulated 200 sets of CD4+ cell count values at seroconversion and rates of CD4+ cell count decline, as follows. Square-root transformed values of the CD4+ cell count at HIV seroconversion and CD4+ cell count slopes in ANRS 1220 Primo-CI cohort participants were fitted with a joint bivariate normal distribution.^15^ This distribution was used to simulate the 200 sets of values for each surveyed individual. Using each set of values, along with CD4+ cell counts measured at ART initiation and Equation (2), we calculated, for each surveyed individual, 200 time intervals between seroconversion and ART initiation. We estimated the average time from seroconversion to ART initiation by averaging the results for each surveyed individual. Finally, we calculated the delay in ART initiation.

The eligibility for ART initiation in Cameroon was raised from 200 to 350 cells/μL in August 2010. We therefore calculated the average time from seroconversion to ART initiation and delay in ART initiation using the estimated average time from seroconversion to 200 cells/μL for individuals who initiated ART before August 2010. For individuals who initiated ART after August 2010, we used the estimated average time from seroconversion to 350 cells/μL.

To assess the robustness of our results, we performed sensitivity analyses which considered two scenarios of HIV virulence. First we assumed higher virulence leading to 5% lower CD4+ cell count at HIV seroconversion and 5% higher CD4+ cell count slope. Second we assumed lower virulence leading to 5% higher CD4+ count at HIV seroconversion and 5% lower CD4+ cell count slope. These assumptions were motivated by findings that showed a 25% decrease in the CD4+ count at seroconversion over more than 20 years,^16^ while our study only considered individuals who initiated ART between 2007 and 2010. We compared the results from the sensitivity analyses with the main estimates using the *Z*-test. All analyses were performed using Stata version 11.1 (StataCorp. LP, College Station, United States of America).

## Results

### Model fitting

By fitting the mixed model to the seroconverter data, we estimated that the mean CD4+ cell count at seroconversion was 539 cells/μL (95% confidence intervals, CI: 516 to 562) and that the mean CD4+ cell decline in the first year after seroconversion was 53 cells/μL (95% CI: 51 to 56). Higher CD4+ cell counts at cohort inclusion and longer intervals from seroconversion to cohort inclusion were associated with higher CD4+ cell counts at seroconversion (*P* < 0.001; Table 3). In addition, higher CD4+ cell counts at cohort inclusion were associated with a more rapid CD4+ cell decline (*P* = 0.009; Table 3). None of the other covariates was significantly associated with the intercept or slope.

**Table 3:** Influence of selected characteristics of ANRS 1220 Primo-CI cohort participants on the CD4+ cell count at seroconversion and its slope, as estimated from the linear mixed model

Effect	Coefficient (95% CI)	*P*
**Fixed effect**		
Square-root CD4+ cell count intercept		
Baseline	12.035 (11.358 to 12.712)	< 0.001^a^
CD4+ cell count at cohort inclusion (per 1 cell/μL increase)	0.020 (0.019 to 0.021)	< 0.001^a^
Time from HIV seroconversion to cohort inclusion (per one-year increase)	0.973 (0.672 to 1.273)	< 0.001^a^
Square-root CD4+ cell count slope (cells/μL decrease per year)		
Baseline	−0.676 (−0.972 to −0.379)	< 0.001^a^
CD4+ cell count at cohort inclusion (per 1 cell/μL increase)	−0.001 (−0.001 to −0.000)	0.009^a^
Time from HIV seroconversion to cohort inclusion (per one-year increase)	−0.065 (−0.186 to 0.056)	0.292^a^
**Random effect**		
Intercept variance	2.007 (1.390 to 2.898)	< 0.001^b^
Slope variance	0.525 (0.394 to 0.701)	
Covariance between intercept and slope	−0.165 (−0.430 to 0.101)	

CI: confidence interval; HIV: human immunodeficiency virus.

^a^ Wald test.

^b^ The likelihood ratio test rejected the null hypothesis that all coefficients were simultaneously equal to 0.

Note: The following variables: age at seroconversion, gender, CD4+ cell count at cohort inclusion and time from seroconversion to cohort inclusion, were initially included in the multivariable analysis. We used a backward stepwise selection procedure in the final model to retain only those covariates significantly associated (*P* < 0.05) with the intercept and slope.

### Time to ART eligibility

We replaced the parameters in Equation (2) with their estimates from the previous step to calculate the time from HIV seroconversion to ART eligibility:
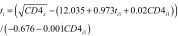
(3)where *CD*4*_x_* is the CD4+ cell count ART eligibility threshold, t*_i_*_1_ is the estimated number of years from seroconversion to cohort inclusion, and *CD*4*_i_*_1_ is the CD4+ cell count at cohort inclusion.

The average times from seroconversion to CD4+ cell counts of less than 350 and  200 cells/μL were 4.0 and 7.0 years, respectively. The corresponding median times were 3.8 and 7.1 years (Table 4).

**Table 4:** Estimated time from HIV seroconversion to ART eligibility

HIV sero-converters	2010 WHO ART eligibility threshold^a^				2006 WHO ART eligibility threshold^b^	
	Mean years (95% CI)		Median years (IQR)		Mean years (95% CI)	Median years (IQR)
All		4.0 (3.7 to 4.3)	3.8 (2.0 to 5.7)		7.0 (6.8 to 7.3)	7.1 (5.2 to 8.8)
Women		4.1 (3.6 to 4.5)	3.9 (2.2 to 5.9)		7.0 (6.6 to 7.4)	7.2 (5.3 to 8.7)
Men		4.0 (3.6 to 4.3)	3.7 (1.9 to 5.7)		7.0 (6.7 to 7.4)	7.1 (5.2 to 8.9)

ART: antiretroviral therapy; CI: confidence interval; HIV: human immunodeficiency virus; IQR: interquartile range; WHO: World Health Organization.

^a^ The threshold is a CD4+ cell count less than 350 cells/μL.

^b^ The threshold is a CD4+ cell count less than 200 cells/μL.

### Cameroon indicators

Women had a higher CD4+ cell count at ART initiation than men (*P* < 0.001). The median CD4+ cell count at ART initiation increased significantly from 123 cells/μL in 2007–2009 to 147 cells/μL in 2010 among men, and from 147 cells/μL in 2007–2009 to 170 cells/μL in 2010 among women.

#### Time to ART initiation

The estimated average time from seroconversion to ART initiation fell from 10.4 years (95% CI: 10.3 to 10.5) in 2007–2009 to 9.8 years (95% CI: 9.6 to 10.0) in 2010 (Table 5); the corresponding median times were 9.9 and 9.2 years. The time was significantly shorter for women than men in 2007–2009 (10.2 versus 10.9 years, *P* < 0.01), while the difference was no longer significant in 2010 (9.7 versus 10.0 years, *P* = 0.36).

**Table 5:** Estimated time from HIV seroconversion to antiretroviral treatment initiation and delay in antiretroviral treatment initiation, Cameroon, 2007–2010

Year of ART initiation	Time from HIV seroconversion to ART initiation		Delay in ART initiation^a^ (95% CI)
	Mean years (95% CI)	Median years (IQR)	
**2007–2009**			
All	10.4 (10.3 to 10.5)	9.9 (7.6 to 13.1)	3.4 (3.1 to 3.7)
Women	10.2 (10.0 to 10.3)	9.7 (7.5 to 12.7)	3.2 (2.8 to 3.7)
Men	10.9 (10.6 to 11.1)	10.6 (7.9 to 13.8)	3.9 (3.5 to 4.3)
**2010**			
All	9.8 (9.6 to 10.0)	9.2 (6.6 to 12.7)	5.8 (5.6 to 6.2)
Women	9.7 (9.4 to 10.0)	9.1 (6.6 to 12.6)	5.6 (5.2 to 6.0)
Men	10.0 (9.6 to 10.5)	9.4 (6.8 to 12.9)	6.0 (5.4 to 6.6)

ART: antiretroviral therapy; CI: confidence interval; HIV: human immunodeficiency virus; IQR: interquartile range.

^a^ The delay in ART initiation was calculated by subtracting the average time from seroconversion to ART eligibility from the average time between HIV seroconversion and ART initiation.

Note: The ART eligibility threshold was 200 cells/μL in 2007–2009 and 350 cells/μL in 2010.

#### Delay in ART initiation

The estimated delay in ART initiation is shown in Table 5. Overall, the delay increased from 3.4 years (95% CI: 3.1 to 3.7) in 2007–2009 to 5.8 years (95% CI: 5.6 to 6.2) in 2010. Women had shorter delays than men (3.2 versus 3.9 years in 2007–2009, and 5.6 versus 6.0 years in 2010).

### Sensitivity analyses

The results from our sensitivity analysis are shown in Table 6. Assuming a 5% increase in HIV virulence led to a 1.1–1.3 year shorter average time from seroconversion to ART initiation, corresponding to an 11–12% decrease (*P* < 0.0001). While assuming a 5% decrease in HIV virulence led to a 1.3–1.4 year longer average time, corresponding to an 11–14% increase (*P* < 0.0001). In contrast, the delay in ART initiation was not significantly changed under the two sensitivity analysis scenarios except for the overall value in 2010 (*P* = 0.002) and the value among women in 2010 (*P* < 0.05), where we found a 9–10 % decrease when assuming a 5% increase in HIV virulence and a 12–13% increase when assuming a 5% decrease in HIV virulence.

**Table 6:** Sensitivity analyses of estimated time from HIV seroconversion to antiretroviral treatment initiation and delay in antiretroviral treatment initiation, Cameroon, 2007–2010

Year of ART initiation	Time from HIV seroconversion to ART initiation		Delay in ART initiation (95% CI)
	Mean years (95% CI)	Median years (IQR)	
**Scenario with increase in HIV virulence^a^**			
2007–2009			
All	9.2 (9.0 to 9.3)***	8.6 (6.5 to 11.6)	3.2 (3.0 to 3.4)
Women	9.0 (8.8 to 9.1)***	8.4 (6.5 to 11.3)	3.0 (2.8 to 3.2)
Men	9.6 (9.4 to 9.8)***	9.2 (6.8 to 12.3)	3.6 (3.4 to 3.8)
2010			
All	8.6 (8.4 to 8.9)***	7.9 (5.6 to 11.3)	5.2 (4.9 to 5.5)*
Women	8.5 (8.2 to 8.7)***	7.8 (5.5 to 11.2)	5.1 (4.8 to 5.4)*
Men	8.9 (8.5 to 9.3)***	8.3 (5.8 to 11.6)	5.5 (5.0 to 6.0)
**Scenario with decrease in HIV virulence^b^**			
2007–2009			
All	11.8 (11.6 to 11.9)***	11.2 (8.7 to 14.8)	3.7 (3.5 to 3.9)
Women	11.6 (11.4 to 11.7)***	10.9 (8.6 to 14.4)	3.5 (3.3 to 3.7)
Men	12.3 (12.0 to 12.6)***	11.9 (9.0 to 15.6)	4.2 (3.9 to 4.5)
2010			
All	11.1 (10.9 to 11.4)***	10.4 (7.5 to 14.4)	6.5 (6.2 to 6.8)**
Women	11.0 (10.7 to 11.3)***	10.2 (7.4 to 14.3)	6.3 (5.9 to 6.7)*
Men	11.4 (10.9 to 11.9)***	10.8 (7.8 to 14.7)	6.8 (6.2 to 7.4)

ART: antiretroviral treatment; CI: confidence interval; HIV: human immunodeficiency virus; IQR: interquartile range; *P < 0.05; **P < 0.02; ***P < 0.0001.

^a^ We assumed 5% lower CD4+ count at HIV seroconversion and 5% higher CD4+ cell count slope.

^b^ We assumed 5% higher CD4+ count at HIV seroconversion and 5% lower CD4+ cell count slope.

## Discussion

Delays in starting ART are lost opportunities for both the individual and the community. Therefore expanding ART programmes and accelerating ART initiation will be important to control the HIV epidemic. Here, we propose a method to estimate two new population-level indicators. Delay in ART initiation quantifies the loss of opportunity for people living with HIV. The time from HIV seroconversion to ART initiation quantifies the number of years that HIV-infected individuals were able to transmit HIV.

Our approach requires two data sets. First, data on the natural history of the CD4+ cell count decline during HIV infection. Second, data on CD4+ cell counts at ART initiation among individuals who are representative of the population of interest. The first data set can be obtained from the cohort of seroconverters in a nearby country if not available in the country of interest. Such cohorts exist in low-, middle- and high-income countries.^17^^,^^18^ The second data set is collected in most countries, as CD4+ cell count is an important parameter for the decisions of ART initiation and clinical management,^19^ even in countries implementing ART initiation regardless of CD4+ count levels. Thus, our approach can be used to estimate the delay in ART initiation in most settings.

Our approach is based on two main assumptions. First, we converted CD4+ cell counts into time since HIV seroconversion. We assumed that individuals with low CD4+ cell counts had been infected with HIV longer than individuals with high CD4+ cell counts. This is a reasonable assumption at the population level. Other studies have used a similar approach to estimate the time from seroconversion to ART eligibility at the population level.^17^^,^^18^ Second, we assumed that all HIV-infected individuals are eligible for ART only when the CD4+ threshold is reached. In reality, some individuals with symptomatic HIV disease are eligible for ART regardless of CD4+ cell count.^3^^–^^5^ Therefore, we may have overestimated the average time from seroconversion to ART eligibility and thus underestimated the delay in ART initiation. We did not account for people that have not yet initiated ART or those who die before ART initiation. Furthermore, our approach does not account for what happens after ART initiation, such as treatment adherence.

In Cameroon, we found that the time between HIV seroconversion and ART initiation fell between 2007–2009 and 2010. However, the delay in ART initiation increased during the same period because the ART eligibility threshold was lowered. We also found that women who started ART in 2007–2009 had shorter delays than men, whereas no significant sex difference was found in 2010. This reflects a small sex difference in the CD4+ cell counts at ART initiation in 2010.

### Limitations

As there was no available cohort of seroconverters in Cameroon, we used data from a cohort in Côte d’Ivoire. HIV type 1 (HIV-1) genetic diversity is high in Cameroon, while in Côte d’Ivoire, the HIV-1 CRF 02_AG variant predominates.^20^^,^^21^ However, a study conducted in Cameroon and Senegal showed no difference in clinical progression between patients infected with CRF 02_AG and patients infected with other variants.^22^ In addition, our estimates of the CD4+ cell count at seroconversion and the CD4+ cell count decline are similar to those obtained in other sub-Saharan countries,^23^ suggesting that any differences in disease progression at the population level would be small. Finally, it is conceivable that HIV has become more or less virulent over the course of the pandemic.^16^^,^^24^^,^^25^ Our sensitivity analyses showed that changes in HIV virulence affected the average time between HIV seroconversion and ART initiation, while the delay in ART initiation was less sensitive to assumptions about such changes.

The results from Cameroon show that some progress has been made in reducing late ART initiation. Changes in ART guidelines and decentralization of HIV care could have affected this decrease. In particular, as new guidelines were introduced, patients in care with CD4+ cell counts above 200 cells/μL could have become eligible for ART and thus received ART earlier. Likewise, decentralization of HIV care could have made ART available earlier at the rural level.^10^ However, the estimated time of approximately 10 years to initiate ART and approximately 6 years for the delay in ART initiation indicate that the progress is insufficient and calls for interventions that accelerate ART initiation in Cameroon.

In conclusion, to optimize the control of HIV infection both at individual and community level, ART initiation will need to accelerate to close the gap between the WHO-recommended and actual timing of ART initiation. The proposed indicators should help monitor whether public health interventions are successful in achieving these goals.
